# Microbial Quality of Water in Rural Households of Ethiopia: Implications for Milk Safety and Public Health

**Published:** 2014-06

**Authors:** Kebede Amenu, Marisa Spengler, Markemann André, Anne Valle Zárate

**Affiliations:** ^1^Institute of Animal Production in the Tropics and Subtropics, University of Hohenheim, Garbenstr. 17, 70599 Stuttgart, Germany; ^2^School of Veterinary Medicine, Hawassa University, PO Box 5, Hawassa, Ethiopia

**Keywords:** *Escherichia coli*, Faecal contamination, Milk, Rural community, Water, Ethiopia

## Abstract

Waterborne pathogenic agents affect the health of people either by direct consumption of contaminated water or by its indirect use in food production and/or processing. Studies on the microbiological quality of water in rural areas of Ethiopia are still limited, especially at the household level. The aim of the present study was to assess the microbial quality of water from different sources in rural households in two districts of the Ethiopian Rift Valley area. The correlation between *E. coli* counts in water and milk was also investigated. In total, 233 water samples (126 collected in dry and 107 in wet season) and 53 milk samples (19 from raw milk and 36 from processed milk products) were analyzed for *E. coli* contamination. The overall prevalence of *E. coli* in water samples was 54.9% (n=233). In most of the analyzed samples, a higher prevalence of *E. coli* was recorded during the wet compared to the dry season. The highest load of *E. coli* was detected in water samples from dugouts. The quality of raw milk and traditionally-processed milk products showed variations between districts, and the traditionally-processed milk products were found to contain higher *E. coli* loads than raw milk. The correlation between the *E. coli* counts in water and milk only showed a weak but positive relationship (*r*=0.1). Taking *E. coli* as a proxy for water quality, the microbiological quality of water consumed in the study area was found to be very poor, posing a potential food safety and health risk to the rural communities.

## INTRODUCTION

Rural households in developing countries, like Ethiopia, commonly depend on water sources that are located at some distance from their homestead, requiring collection, transportation, and storage before being used (1). Under such circumstances, the microbial quality of water destined for various domestic uses is affected by both quality status at the source and the handling practices of water during collection, transportation, and storage (2,3). Consequently, the health benefits associated with improvements in water supply depend on the quality of water at the source and the point-of-consumption (4). Water samples taken from household storage-vessels might implicate an actual risk of consumers’ getting exposed to microbial agents, with subsequent potential health impacts (3,5).

In many developing countries, microbial contamination of water is causing various diseases (6). Children, women, immunocompromised individuals, and rural residents are considered to be at the highest risk of contracting waterborne pathogenic microorganisms (7). People can become infected by waterborne pathogenic agents, if they either consume contaminated water directly or indirectly through its use in food production, processing, or preparation (8). Milk products are specifically prone to bacterial contamination, and a broad range of pathogens causing human diseases are milkborne (9). Because contaminated water is a potential source of milk contamination, it has been suggested that water used during the processing of milk and for cleaning the equipment should have quality standards equivalent to that of drinking-water (10). However, people in rural parts of developing countries, in particular sub-Saharan Africa, may not have access to improved water sources and are forced to use water from contaminated sources, thus compromising the safety of milk and milk products.

Among several microbial quality indicators of faecal contamination in water and food, *Escherichia coli* represent a very specific and well-accepted one. The detection of *E. coli* in water or food indicates presence of other dangerous pathogenic microorganisms, specifically those responsible for gastrointestinal illness (11).

Studies on the microbiological quality of water in rural areas of Ethiopia are limited and mainly focused on urban settings (12-14). The present study aimed to assess the microbial quality of water used by rural households for domestic purposes (primarily for drinking), based on *E. coli* as a quality indicator. Special emphasis was given on the association between the quality of water destined for domestic use and the quality of milk. This was done to assess the potential health risk associated with low microbiological quality of water intended for drinking and domestic use, including the cleaning of milking utensils, milk storage and processing. It was hypothesized that the quality of water consumed in rural communities of the study area is poor, posing a potential health risk to people. It was further hypothesized that the quality of water intended for drinking is affected by season and the type of water source from which it was initially collected. Finally, the microbiological quality of milk may pose further risks to human health and may be affected by poor water quality.

## MATERIALS AND METHODS

### Study area

The study was carried out in Lume and Siraro districts of Oromia Regional State located in the Rift Valley area of Ethiopia. Mojo and Lokke, the respective administrative centres of Lume and Siraro districts, are situated respectively 70 km and 308 km south of Addis Ababa. According to unpublished secondary data obtained from the District Administration Offices, the population in Lume was estimated at 126,933 in 2010, with 67% living in rural areas, following a clustered settlement pattern. In Siraro, the estimated population was 167,932, with 84% living in rural areas in dispersed settlements. The proportion of the population in both the districts with access to improved water sources for domestic use was low, with a rural coverage of improved water sources of 38.6% in Lume and 14% in Siraro (based on secondary data obtained from the Rural Water Development Offices of respective districts).

More than 98% of the dairy cattle kept in the study area are Zebu breeds, the remainder being Holstein or Jersey crossbreds. Farmers keeping crossbred dairy cattle sell surplus milk to primary milk marketing cooperatives, private milk collectors, hotels, or neighbours. Milk from farmers keeping Zebu cattle is entirely destined for home consumption, with the exception of butter and soft cheese (*ayib*) that is sold to local markets or neighbours providing a supplemental source of income.

### Sampling scheme

A total of 233 water samples were collected in the months of December 2010 to January 2011 and July to August 2011, corresponding to dry (n=126) and wet (n=107) season respectively. A total of 53 milk samples were also collected during the wet season. All samples were taken from randomly-selected households in two districts of the Rift Valley area, Lume, and Siraro. Initially, 160 households were targeted to be surveyed (repeatedly in the dry and wet seasons). However, water samples could not be collected from 34 households in the dry season and 53 households in the wet season because the households did either have no water at the time of the visit or access to the homesteads was not possible. Failure to get the samples was due to the fact that drinking-water fetched from distant sources was quickly used by family members, and the households were left without water for several hours of the day. Moreover, in the rainy season, some of the homesteads were difficult to access for causes associated with flooding of the area and subsequent inconvenience for transportation. Table 1 shows the number of water samples fetched by the initial water sources by district and season.

Milk and milk products could only be collected from a subsample of households since several farmers did not have lactating cows, or milk was destined solely for calves. Accordingly, 14 and 22 raw milk samples from household containers and 5 and 12 samples from processed milk products (e.g. yoghurt and skim milk) were collected in Lume and Siraro districts respectively.

### Sample processing

The water samples were analyzed for the presence of *E. coli* within 12 hours after collection. A commercially available chromogenic agar medium (Brilliance^TM^ *E. coli*/coliform selective agar, Oxoid CM 1046) was used for the enumeration of *E. coli* in the samples. Ringer's solution (Oxoid) was used for diluting water samples (dilution factors: 10^1^, 10^2^, and 10^3^) to get a countable number of colonies per plate. The diluted samples were inoculated on the agar and incubated for 24 hours at 37 °C as recommended by the manufacturer. Membrane filtration was applied for the selected water samples that were assumed to contain low bacterial indicators (samples from boreholes). From the chromogenic agar plates, purple colonies were counted and recorded as *E. coli*. Results were obtained by multiplying the counted colonies with the dilution factor and then expressed as colony-forming units (CFU) per 100 mL of water.

For the milk samples, serial dilutions (10^1^ to 10^4^) were prepared, and the diluted samples were cultured using the pour plate technique. After incubating the cultured samples for 24 hours at 37 °C, purple-coloured colonies were counted similar to the water samples, and then expressed as CFU/mL of milk.

### Statistical analysis

Percentages of households that had water from different sources at the time of visits were compared by district between seasons using McNemar's test. The prevalence of *E. coli* contamination of the water samples was calculated by dividing the number of samples with counts greater than zero CFU/100 mL by the number of samples analyzed. The non-parametric Wilcoxon signed-rank test was used for comparing *E. coli* counts of the different water sources between the dry and wet season. The Mann-Whitney U-test was used in assessing the equality of microbial load between raw milk and processed milk products, which were compared by district. The relationship between *E. coli* counts of milk and water for the wet season data was assessed by calculating the Spearman's rank correlation coefficient. SAS 9.1 (SAS Institute Inc., Cary, North Carolina, USA) was used for all statistical analyses.

## RESULTS

### Water sources and microbiological quality

Water sources for domestic uses comprised hand-dug wells, boreholes, dugouts, springs, and rainwater collected from roof. Statistically significant seasonal differences between seasons were found in the percentage of households obtaining water from the different sources only in Siraro, but not in Lume, as indicated in Table 1. From the total households visited in Lume, 64.9% and 72.1% fetched water for domestic consumption from boreholes in the dry and wet season respectively. In Siraro, a similar percentage of households (72.5%) obtained water from boreholes in the dry season but only 20.4% in the wet season. A large proportion of households shifted from boreholes to dugouts and rainwater collected from roof in the wet season (Table 1).

The overall prevalence of *E. coli* contamination in water samples (>0 CFU/100 mL of water) was 54.9% (n=233). The aggregated wet and dry season data showed that 34.1% (n=129) water samples of the boreholes, 95.4% (n=43) of the dugouts, 76.5% (n=17) of the hand-dug wells, 72.4% (n=29) rainwater collected from roof, and 60% (n=15) of the springs were contaminated with *E. coli*. In most of the water samples analyzed, a higher prevalence of *E. coli* was generally recorded during the wet season compared to the dry season (Table 2).

*E. coli* loads showed significant differences between the dry and the wet season for all water sources, except for hand-dug wells (Table 3). As indicated in Table 3, the lowest concentration of *E. coli* counts was detected in borehole water samples (also low variation, interquartile range=0 CFU in both seasons) while the highest values were found in water from dugouts (high variation, interquartile range=1,300 CFU in dry and 33,000 CFU in wet season).

**Table 1.** Percentage of water samples collected from households by district, season, and water source

District	Water source	Dry season	Wet season	Statistical significance
Lume		n=57	%	n=53	%	
	Borehole	37	64.9	31	72.1	ns
	Hand-dug well	11	19.3	6	14.0	ns
	Spring	9	15.8	6	14.0	ns
Siraro		n=69		n=54		
	Borehole	50	72.5	11	20.4	*
	Dugouts	13	18.8	30	55.6	*
	Roof rainwater	6	8.7	23	42.6	*

*Statistically significant between seasons (McNemar's test, p<0.05); ns=Not significant

**Table 2.** Prevalence of *E. coli* in water destined for human consumption by district, source, and season

District	Water source	Dry season	Wet season
		n	%	n	%
Lume	Borehole	37	24.3	31	61.3
	Hand-dug well	11	72.7	6	83.3
	Spring	9	90.0	6	16.7
Siraro	Borehole	50	22.0	11	45.5
	Dugouts	13	100.0	30	93.3
	Rainwater from roof	6	50.0	23	78.3

### Microbiological quality of milk and milk products

The prevalence of *E. coli* in raw milk samples from household containers was 21.4% (n=14) in Lume and 54.6% (n=22) in Siraro. For the traditionally-processed milk products, the prevalence of *E. coli* was 60% (n=5) in Lume and 50% (n=12) in Siraro. Though not statistically significant (Mann-Whitney U-test), p>0.05), the *E. coli* counts in raw milk from household containers were higher in Siraro (mean=1,068, median=15, interquartile range=1,000) than in Lume (mean=360, median=0, interquartile range=0). On the other hand, the samples of the processed milk products in Lume were found to contain higher *E. coli* counts (mean= 2,880, median=400, interquartile range=4,000) compared to those in Siraro (mean=1,595, median=20, interquartile range=950); the difference in counts between the districts was found to be statistically significant (Mann-Whitney U-test, p<0.05). It was evident that, in both the districts, the traditionally-processed milk products contained higher *E. coli* counts compared to raw milk samples from household containers.

The correlation between the *E. coli* counts of water and milk showed only a weak but positive relationship (Spearman's rank correlation coefficient *r*=0.1, p=0.5, n=53).

## DISCUSSION

The use of different water sources for domestic consumption, irrespective of the quality, indicates that people in the investigated districts do not have sufficient access to reliable drinking-water. During the wet season, a considerable number of farmers in Siraro switched from boreholes to dugouts and rainwater collected from roof for domestic use (Table 1), both being highly-contaminated water sources (Table 2 and 3). Such intermittent use of potable and non-potable water sources has negative impacts on community health. It has been indicated that a repeated low-dose exposure to pathogens can reduce the incidence of waterborne diseases in communities that are permanently depending on non-potable water sources compared to intermittent users (15). In developing countries, frequent dysfunction of water supply schemes present major challenges in ensuring a sustainable provision of drinking-water services (16). In this regard, Hunter *et al*. (17) indicated that only a few days of interrupted water supply can be sufficient to destroy the health benefits from the provision of clean drinking-water when communities shift to contaminated water sources after a period of using potable water. The improved water schemes of the study area were also highly unreliable with frequent breakdowns, and the community reverted to low-quality water sources, with potentially high burdens on human health (18).

The microbiological safety of food and water is commonly assessed by quantifying bacterial indicators because of the difficulty in assessing all potentially pathogenic microorganisms. Total coliforms and *E. coli* are the most common indicator bacteria for such assessments, with *E. coli* being the more specific one for water or food contaminated with animal or human faecal materials (19,20). The presence of coliform bacteria other than *E. coli* in drinking-water indicates inefficient water treatment schemes (if present). In the present study, water sources utilized by the communities were largely untreated. The World Health Organization recommends that water directly intended for human consumption be free from *E. coli* contamination (11) since the presence of *E. coli* indicates a potential health risk for consumers.

In general, the microbial quality of water at the point-of-consumption is influenced by many factors, such as the initial state of contamination of water at sources, the storage conditions, fetching and handling practices as well as the applied treatment methods to improve quality (1,2). The high prevalence of *E. coli* found in water samples from dugouts in the present study might be attributed to the initial contamination by human and animal activities around the source. Similar studies found borehole water sources to be free from faecal indicators, i.e. *E. coli* at source level (18,21). However, in the present study, more than 20% and 45% of the water samples from boreholes in the dry and the wet season respectively were contaminated at the point-of-consumption (Table 2). This could be related to overcrowded borehole pumps (public standpipes) with people and animals during water collection, loose plastic hose-fittings to the borehole pipe and generally poor handling practices during collection, transportation, and storage. Another source of considerable concern regarding the high microbial contamination of water could be the bacterial biofilm formation on the walls of the containers due to inadequate cleaning after each use, or the recurrent use of the same containers for fetching water from different sources. Thus, the higher prevalence of *E. coli* in the wet season compared to the dry season could be associated with such biofilm production.

**Table 3.** Seasonal differences in *E. coli* counts (CFU/100 mL) in water from household containers by district and source

District	Water source	Dry season								Wet season						Statistical significance
		n	Mean	Median	IQR	Min	Max	%<IQR	n	Mean	Median	IQR	Min	Max	%<IQR	
Lume	Borehole	37	684	0	0	0	16,800	0.0	31	710	100	500	0	11,400	74.2	*
	Hand-dug well	11	275	100	200	0	1,700	63.6	6	1,100	800	1,800	0	3,000	66.7	ns
	Spring	9	2,855	200	700	0	21,600	66.7	6	17	0	0	0	100	0.0	*
																
Siraro	Borehole	50	242	0	0	0	4,100	0.0	11	100	0	200	0	400	72.7	*
	Dugouts	13	3,177	500	1,300	0	26,400	69.2	30	82,036	6,000	33,000	0	500,000	73.3	*
	Roof rainwater	6	83	50	200	0	200	66.7	23	43,359	450	2,100	0	1,340,000	69.6	*

*Statistically significant between seasons (Wilcoxon signed-rank test, p<0.05); %<IQR=Percentage of samples with *E. coli* counts below IQR; IQR=Interquartile range; Max=Maximum; Min=Minimum; ns=Not significant

Rainwater collected from roof is generally assumed to be safe and potable (22). In this study, 50% and 78.3% of rainwater samples were contaminated with *E. coli* in the dry and wet season respectively. These relatively high contamination rates are most likely associated to the improper design of the rainwater harvesting system, which consisted of concrete cisterns or temporary containers (buckets or pots). In most cases, the concrete cisterns were either open or barely covered, and the temporary containers to harvest rainwater were not put high enough above the ground to protect soil contamination.

The high *E. coli* contamination of water destined for human consumption recorded in the present study has implications for the efforts being undertaken to provide improved drinking-water resources. The improvements of water resources may be compromised, unless they are accompanied with proper health education, especially for women since they are generally responsible for the collection and handling of water for domestic consumption.

In the present study, traditionally-processed dairy products showed higher *E. coli* counts compared to raw milk. In contrast to this, Mhone *et al*. (23) recorded lower counts of *E. coli* in processed dairy products than in raw milk samples. It is assumed that the organic acids produced as a result of natural fermentation of milk and milk products can potentially reduce the growth of microbial agents, including *E. coli* (24). However, since consumption of the milk products usually takes place before the fermentation is completed, this fermentation cannot be a guarantee in reducing associated health risks (25). The higher *E. coli* counts in the processed milk products in Lume compared to Siraro could be partly attributed to differences in the smoke treatment of milk utensils. During collection of samples from the field, it was observed that utensils for storing and handling of milk were more commonly smoked by households in Siraro compared to those in Lume. The practice of smoking the vessels used for the storage of milk is a common practice in various parts of Ethiopia (26). Various plant species are used in central Ethiopia for the smoking treatment of milk vessels and milk containers by smallholder farmers, the most common one being the wild olive or ‘*Ejersa*’ (*Olea europaea* subspecies *africana*) (27). During the smoking process, wood chips of the plants are burnt, introduced into the vessel, and whirled inside for some minutes with the lid of the vessel closed. In other cases, the vessel is inverted over the smoking chips until the smoke dies out (26). In a laboratory experiment, Ashenafi (28) showed that the smoking of milk containers slows the growth of coliforms and lactic acid bacteria, thus contributing to flavour, safety, and quality of the finished products. Irrespective of the traditional smoking and fermentation practices, the high bacterial counts found in both raw milk and processed milk products in the present study are a foreseeable health risk for consumers in the study area.

The correlation of *E. coli* counts between milk and water in this study was low and not significant, probably owing to the small number of paired samples. In contrast, Kivaria *et al*. (29) reported a significant influence of the bacterial quality of water on the total bacterial counts in milk in smallholder dairy systems. Another study conducted in intensive dairy production also showed a significant effect of the quality of washing-water used, or the cleaning of milking equipment on the bacteriological quality of raw milk (30). Although no statistically significant correlation was found in the present investigations, poor-quality water as a source of bacterial contamination in smallholder dairy production systems should not be underestimated. The water samples analyzed in the current study were actually those intended for direct human consumption but it can be assumed that more water of inferior quality is used for sanitation purposes in different domestic activities, including milk processing.

The detection of *E. coli* in water and milk highly suggests the presence of dangerous pathogens, such as *Vibrio cholerae*, *Salmonella* Typhi, *Salmonella* Paratyphi and *Campylobacter* spp. (31). Some strains of *E. coli* (e.g. *E. coli* O157:H7) are causing waterborne or foodborne illnesses with mild to severe symptoms (32,33). The severe consequences of infection with *E. coli* serotype 0157:H7 include gastroenteritis, haemorrhagic colitis, haemolytic-uraemic syndrome (HUS), and thrombotic thrombocytopenic purpura (33,34).

### Conclusions

Taking *E. coli* as a proxy for the presence of enteric pathogens in water, it can be concluded that the microbiological quality of water consumed in the study area was found to be rather poor, posing a potential food safety and health risk to the rural communities. It is further concluded that the microbiological quality of water varied according to season and type of water sources. High *E. coli* counts were recorded during the wet season compared to the dry season. The highest load of *E. coli* was detected in water samples initially fetched from dugouts. Although the microbiological quality of boreholes (the most common improved water sources in the study area) at the point-of-collection was good (18), more than 20% of the dry season samples and 45% of the wet season samples were found to be contaminated with *E. coli* at the point-of-consumption (household containers), putting the health of consumers at risk. The results of the present study revealed a high post-collection recontamination of water associated with mishandling and improper storage. Recontamination of water can severely compromise the expected health benefits from the installation of improved water sources. Despite a non-significant correlation between *E. coli* counts in milk and water, the level of recontamination of water and direct contamination of milk depends on multiple factors, which can only be revealed by site-specific studies with a higher number of paired samples. Still, the assumption holds that the recorded poor water quality contributes to the low microbiological quality and safety of milk and dairy products produced and consumed in the area but further research is required for more valid inferences. Moreover, further action in the improvement of water supply schemes in the area and awareness creation on safe water handling practices are necessary.

## ACKNOWLEDGEMENTS

The authors would like to acknowledge the German Academic Exchange Service (DAAD) for funding the research. The study also benefited from partial financial support of the ‘Safe Food-Fair Food’ project, a collaborative research project led by the International Livestock Research Institute (ILRI), Kenya, and funded by *Bundesministerium für wirtschaftliche Zusammenarbeit und Entwicklung* (BMZ), Germany.

## References

[1] Wright J, Gundry S, Conroy R (2004). Household drinking water in developing countries: a systematic review of microbiological contamination between source and point-of-use. Trop Med Int Health.

[2] Trevett AF, Carter RC, Tyrrel SF (2005). The importance of domestic water quality management in the context of faecal-oral disease transmission. J Water Health.

[3] Rufener S, Mäusezahl D, Mosler H-J, Weingartner R (2010). Quality of drinking-water at source and point-of-consumption—drinking cup as a high potential recontamination risk: a field study in Bolivia. J Health Popul Nutr.

[4] Oswald WE, Lescano AG, Bern C, Calderon MM, Cabrera L, Gilman RH (2007). Fecal contamination of drinking water within peri-urban households, Lima, Peru. Am J Trop Med Hyg.

[5] Moyo S, Wright JA, Ndamba J, Gundry SW (2004). Realising the maximum health benefits from water quality improvements in the home: a case from Zaka district, Zimbabwe. Phys Chem Earth.

[6] Marino DD (2007). Water and food safety in the developing world: global implications for health and nutrition of infants and young children. J Am Diet Assoc.

[7] Obi CL, Onabolu B, Momba MNB, Igumbor JO, Ramalivahna J, Bessong P (2006). The interesting cross-paths of HIV/AIDS and water in Southern Africa with special reference to South Africa. Water SA.

[8] Kirby RM, Bartram J, Carr R (2003). Water in food production and processing: quantity and quality concerns. Food Control.

[9] Oliver SP, Jayarao BM, Almeida RA (2005). Foodborne pathogens in milk and the dairy farm environment: food safety and public health implications. Foodborne Pathog Dis.

[10] Chye FK, Abdullah A, Ayob MK (2004). Bacteriological quality and safety of raw milk in Malaysia. Food Microbiol.

[11] World Health Organization (2011). Guidelines for drinking water quality.

[12] Admassu M, Wubshet M, Gelaw B (2004). A survey of bacteriological quality of drinking water in North Gondar. Ethiop J Health Dev.

[13] Kifle S, Gadisa T (2006). Microbial quality of Jimma water supply. Ethio J Educ Sci.

[14] Biadglegne F, Tessema B, Kibret M, Abera B, Huruy K, Anagaw B (2009). Physicochemical and bacteriological quality of bottled drinking water in three sites of Amhara Regional State, Ethiopia. Ethiop Med J.

[15] Frost FJ, Roberts M, Kunde TR, Craun G, Tollestrup K, Harter L (2005). How clean must our drinking water be: the importance of protective immunity. J Infect Dis.

[16] De Palencia AJF, Pérez-Foguet A (2012). Quality and year-round availability of water delivered by improved water points in rural Tanzania: effects on coverage. Water Policy.

[17] Hunter PR, Zmirou-Navier D, Hartemann P (2009). Estimating the impact on health of poor reliability of drinking water interventions in developing countries. Sci Total Environ.

[18] Amenu K, Markemann A, Valle Zárate A (2013). Water for human and livestock consumption in rural settings of Ethiopia: assessments of quality and health aspects. Environ Monit Assess.

[19] World Health Organization (1997). Guidelines for drinking water quality.

[20] Byamukama D, Mach RL, Kansiime F, Manafi M, Farnleitner AH (2005). Discrimination efficacy of fecal pollution detection in different aquatic habitats of a high-altitude tropical country, using presumptive coliforms, *Escherichia coli*, and *Clostridium perfringens* spores. Appl Environ Microbiol.

[21] Parker AH, Youlten R, Dillon M, Nussbaumer T, Carter RC, Tyrrel S (2010). An assessment of microbiological water quality of six water source categories in north-east Uganda. J Water Health.

[22] Nevondo TS, Cloete TE (1999). Bacterial and chemical quality of water supply in the Dertig village settlement. Water SA.

[23] Mhone TA, Matope G, Saidi PT (2011). Aerobic bacterial, coliform, *Escherichia coli* and *Staphylococcus aureus* counts of raw and processed milk from selected smallholder dairy farms of Zimbabwe. Int J Food Microbiol.

[24] Ashenafi M (1994). Fate of Listeria monocytogenes during the souring of Ergo, a traditional Ethiopian fermented milk. J Dairy Sci.

[25] Tsegaye M, Ashenafi M (2005). Fate of *Escherichia coli* O157:H7 during the processing and storage of Ergo and Ayib, traditional Ethiopian dairy products. Int J Food Microbiol.

[26] Gonfa A, Foster HA, Holzapfel WH (2001). Field survey and literature review on traditional fermented milk products of Ethiopia. Int J Food Microbiol.

[27] Mekonnen H, Lemma A (2011). Plant species used in traditional smallholder dairy processing in East Shoa, Ethiopia. Trop Anim Health Prod.

[28] Ashenafi A (1996). Effect of container smoking and incubation temperature on the microbiological and some biochemical qualities of fermenting Ergo, a traditional Ethiopian sour milk. Int Dairy J.

[29] Kivaria FM, Noordhuizen JP, Kapaga AM (2006). Evaluation of the hygienic quality and associated public health hazards of raw milk marketed by smallholder dairy producers in the Dar es Salaam region, Tanzania. Trop Anim Health Prod.

[30] Perkins NR, Kelton DF, Hand KJ, MacNaughton G, Berke O, Leslie KE (2009). An analysis of the relationship between bulk tank milk quality and wash water quality on dairy farms in Ontario, Canada. J Dairy Sci.

[31] Ashbolt NJ (2004). Microbial contamination of drinking water and disease outcomes in developing regions. Toxicology.

[32] Griffin PM, Tauxe RV (1991). The epidemiology of infections caused by *Escherichia coli* O157:H7, other enterohemorrhagic *E. coli*, and the associated hemolytic uremic syndrome. Epidemiol Rev.

[33] Olsen SJ, Miller G, Breuer T, Kennedy M, Higgins C, Walford J (2002). A waterborne outbreak of *Escherichia coli* O157:H7 infections and hemolytic uremic syndrome: implications for rural water systems. Emerg Infect Dis.

[34] Wells JG, Shipman LD, Greene KD, Sowers EG, Green JH, Cameron D (1991). Isolation of *Escherichia coli* serotype O157:H7 and other Shiga-like-toxin-producing *E. coli* from dairy cattle. J Clin Microbiol.

